# Research progress on rehabilitation programs for dysphagia in patients with ischemic stroke: A Narrative review

**DOI:** 10.12669/pjms.41.7.9657

**Published:** 2025-07

**Authors:** Wenbin Jiang, Junhe Chen, Ting Xue, Yongmei Jiang

**Affiliations:** 1 Wenbin Jiang, The Affiliated Hospital of Qingdao University, Qingdao City, Shandong Province, China; 2 Junhe Chen, School of Pharmacy, Qingdao Medical College, Qingdao University, Qingdao City, Shandong Province, China; 3 Ting Xue, The Affiliated Hospital of Qingdao University, Qingdao City, Shandong Province, China; 4 Yongmei Jiang, The Affiliated Hospital of Qingdao University, Qingdao City, Shandong Province, China

**Keywords:** Dysphagia, Ischemic stroke, Nursing, Recovery treatment, Thrombus

## Abstract

This review aims to explore the various symptoms caused by ischemic stroke and summarize current clinical care and rehabilitation treatment options for post-stroke dysphagia. Stroke is the second leading cause of death and a leading cause of long-term disability worldwide, with the highest prevalence in developing countries. Among them, ischemic stroke is the most common type, accounting for more than 80% of the total number of strokes and is the focus of current stroke research. More than one-third of ischemic stroke patients will be affected by post-stroke functional impairments, including but not limited to claudication, upper limb dysfunction, visual impairment, swallowing dysfunction, etc. Swallowing disorders refer to symptoms such as pain, weakness in chewing, prolonged swallowing time, choking when eating or drinking, and coughing due to abnormal function of the swallowing center and motor-sensory pathways and reduced control of the oral and throat muscles.

Severe dysphagia will not only affect the patient’s nutritional intake and cause malnutrition, but may also cause serious complications such as dehydration, aspiration pneumonia, and suffocation, leading to poor prognosis and even death. Therefore, the care and rehabilitation treatment of such patients have important clinical significance. The treatment strategies integrated in this review provide a variety of approaches for the rehabilitation of patients with post-stroke dysphagia, emphasizing the importance of personalized medicine and the necessity of multidisciplinary collaboration. Future studies should establish standardized research designs and evaluation indicators to improve the reproducibility of the research and the reliability of the results, hoping to provide more references for the treatment of swallowing dysfunction and the improvement of subsequent clinical care programs.

## INTRODUCTION

Stroke is the second leading cause of death in the world, affecting at least 15 million people around the world every year, one-third of whom die and one-third suffer permanent physical disabilities. Statistics show that the male stroke incidence rate is approximately 62.8 per 100,000 people, while the female stroke incidence rate is 59 per 100,000 people. The incidence of stroke in men is significantly higher than that in women, which may be due to the influence of bad lifestyle habits such as smoking, diet, and drinking. Research shows that the number of strokes is proportional to age, with nearly 75% of strokes occurring in patients over 64 years old.^1^ Nowadays, about 30% of cases occur in young people, which shows that stroke is not only a disease of the elderly in the traditional sense but is also developing among younger people. Usually, this kind of testing only involves elderly subjects. Statistics from the American Heart Association found that women over the age of 75 are more likely to have a stroke than men.^2^

Stroke is usually divided into two main categories: hemorrhagic stroke and ischemic stroke. Hemorrhagic stroke is characterized by the presence of blood in the brain parenchyma that can accumulate and compress adjacent parenchyma. In contrast, ischemic stroke, caused by a blood clot that interrupts blood flow to the brain, accounts for 87% of all stroke cases.^3^ Cardiac embolism, cerebral circulation atherosclerosis, and small vessel occlusion are the main causes of ischemic stroke. Approximately 45% of ischemic strokes are caused by thrombus in small or large arteries, and cardioembolic strokes account for 14-30% of all cerebral infarctions. Another important subtype is the null subtype, which accounts for 15-25% of all ischemic strokes.^4^,^5^

Few neurological diseases are as complex and devastating as stroke, which was once thought to be solely a vascular disorder. However, more and more subsequent studies have proven that the biological processes behind stroke are driven by the interaction of neurons, glial cells, vascular cells, and matrix components. This review aims to explore the various symptoms caused by stroke and their molecular mechanisms, and summarize the current clinical care and rehabilitation treatment options for dysphagia after stroke.

## METHODS

Literature searches were conducted in the PubMed, Web of Science and Google Scholar. In addition to earlier published articles, the latest research published in March 2025 is also cited. We cited literature from around the world, including papers published in Pakistani journals. The keywords used were as follows: stroke, ischemic stroke, dysphagia, nursing, physical impairment, limb disorder, recovery treatment, thrombus, thrombosis, rehabilitation. After reading the titles and abstracts of all articles and eliminating duplicate articles and articles with low relevance, we selected 45 articles for this review. And we also consulted references in relevant articles to ensure coverage was comprehensive and unbiased.

## RESULTS

### The pathogenesis of ischemic stroke:

**Fig.1:**
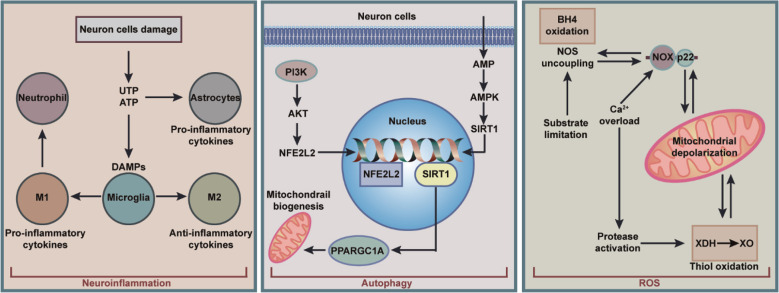
The pathogenesis of ischemic stroke.

The main characteristic of ischemic stroke is its extremely high morbidity, mortality and disability rates.^6^ Among them, acute ischemic stroke accounts for 87% of the total incidence. The principle is that the occlusion of local arteries in the brain tissue leads to the interruption of oxygen and blood supply. Disruption of the blood-brain barrier is one of the hallmark pathophysiological features of ischemic stroke, resulting in increased vascular permeability due to degradation of tight junctions and enhanced endothelial vesicle trafficking. The uncontrolled influx of blood-borne cells, macromolecules, and fluids leads to cytotoxicity and vasogenic edema with serious consequences. Therefore, exploring the pathogenesis of ischemic stroke will help with subsequent research on targeted prevention and treatment. (Fig.1. The pathogenesis of ischemic stroke).

### Neuroinflammation:

Nerve stimulation that occurs in brain tissue is one of the main factors that triggers stroke.^7^ The process of neuroinflammation depends on nerve damage. Ischemic stroke causes necrosis and apoptosis in the brain. All these trigger inflammatory responses associated with reactive oxygen species, chemokines, and cytokines. This process involves a variety of cells that contribute to neuronal death, such as microglia and lymphocytes. Brain damage caused by neuroinflammation exacerbates the release of cytokines in brain tissue and peripheral blood.

These cytokines play an important role in the immune-inflammatory response induced by stroke and have an impact on the progression of cerebral infarction as well as the severity and outcome of the disease.^8^-^10^ Brain cells produce three major cytokines, namely tumor necrosis factor (TNF-α), interleukin (IL)-1 and IL-6, after being subjected to various stimuli such as ischemia. Neurons, astrocytes, microglia, and oligodendrocytes can produce inflammatory mediators, and cytokine receptors are expressed throughout the central nervous system (CNS).

Cytokines are involved in nearly every aspect of stroke, and they have multiple proinflammatory and procoagulant effects on endothelial cells. Cytokines have diverse roles in the brain, and they regulate and mediate local changes caused by inflammation and injury in the central nervous system.^11^ The expression of TNF-α after ischemic stroke will stimulate the expression of leukocyte tissue factors and adhesion molecules, and release IL-1β, nitric oxide, platelet activating factor and endothelin. Inhibits thrombomodulin-related systems. Tissue plasminogen activator is reduced, and plasminogen activator inhibitor-1 is released. Research on the role of IL-1β in the brain initially focused on its role in immune responses. IL-1β can inhibit, exacerbate, or induce neuronal damage and death. Cytokine levels in plasma vary in each diagnostic subtype of ischemic stroke and are closely related to prognosis.

### Autophagy:

Moderate induction of autophagy may serve as a pro-survival mechanism in neuronal cells to sequester degraded or aged organelles, minimal proteins, and cellular components.^12^-^14^ During this process, cells are enveloped by a transient double-membrane structure called the phagosome, which then swells and matures into double-membrane autophagosomes. The formed autophagosomes subsequently fuse with lysosomes, leading to breakdown of cellular contents and recycling of the autophagic matrix building blocks.^15^,^16^ Neurons are particularly susceptible to a variety of intrinsic and extrinsic influences. Under these stressful conditions, autophagy is activated to varying degrees by stress, by maintaining neuronal homeostasis, clearing protein aggregates and damaged mitochondria, and by recycling Amino acids, fatty acids, and glucose maintain energy balance, as well as relieve endoplasmic reticulum (ER) stress, ensuring neuronal cell viability and overall central nervous system health.^17^

Studies have found that excessive induction of autophagy may be to restore cellular homeostasis under lethal stress rather than directly inducing cell death.^18^ Although multiple mechanisms may lead to cell death from excessive autophagy, the duration and conditions of autophagy required to induce cell death remain elusive.^19^ Autophagy-related protein family proteins regulate two different types of autophagy. Existing research has identified more than 30 autophagy-related proteins, which regulate different autophagy pathways in different ways.^20^ To regulate autophagy according to different stress properties, various molecules form a complex signaling pathway network.

In the context of ischemic stroke, cerebral perfusion causes nutrient deprivation, oxidative stress, and ER stress, all of which lead to the induction of autophagy in neuronal cells. Studies have shown that in the case of cardiac ischemia, basal autophagy can protect myocardium and cardiomyocytes from ischemia-induced damage and apoptosis.^21^ Furthermore, induction of basal autophagy is a cytoprotective mechanism that alleviates Parkinson’s disease, Huntington’s disease, and Alzheimer’s disease.

The role of basal autophagy in the pathogenesis and treatment of cardiovascular, metabolic, neurodegenerative, and infectious diseases. However, many ischemic stroke patients face severe brain damage problems due to reperfusion injury and rebleeding complications. Ischemic stroke often triggers maladaptive autophagy that accompanies the postischemic period. Preischemic intervention is effective in counteracting postischemic maladaptive autophagy and neural damage and affects the nature of autophagy in the postischemic period.^22^ Therefore, exploring autophagy in neural tissue in the face of ischemia provides new ideas for preventing and treating stroke.

Many vascular risk factors increase the production of reactive oxygen species (ROS) and promote systemic and cerebrovascular inflammation. The major vascular source of ROS is the uncoupling of superoxide-generating enzymes and nitric oxide synthase (NOS), which generates superoxide instead of NO.^23^ Many of the damaging effects of oxidative stress on blood vessels are related to the biological inactivation of NO by the free radical superoxide, which reduces the bioavailability of NO and prevents its beneficial effects.^24^ Loss of the vasomodulatory role of endothelial NO results in vasoconstriction and reduced NO-dependent vascular responses, negatively affecting the regulation of microvascular blood flow. Loss of various actions of NO promotes platelet aggregation, leukocyte adhesion to endothelial cells, and smooth muscle proliferation, which are critical steps in vascular inflammation.

ROS can directly promote the activation of neuroinflammation by increasing blood-brain barrier permeability by upregulating vascular endothelial growth factor (VEGF) and inducing the expression of cytokines, matrix remodeling enzymes and pro-inflammatory genes through NF-κb.^25^ Among them is an NF-κb-dependent gene product, inducible nitric oxide synthase, which produces large amounts of NO and alters vascular structure and function through nitration and nitrosylation of key proteins. Peroxynitrite, a potent nitrifier produced by the reaction of nitric oxide with superoxide, alters vasomotor responsiveness by inactivating key enzymes, leading to ATP depletion and vascular ion channel dysfunction.^24^

While ROS may set the stage for inflammation, vascular inflammation in turn leads to ROS production, exacerbating vascular damage. Activation of the plasminogen system by oxidative stress and inflammation can promote matrix remodeling, smooth muscle cell migration, and intimal hyperplasia. These factors promote atherosclerosis and changes in vascular structure.^26^ Oxidative stress is also the main way in which risk factors have harmful effects on blood vessels.

### Functional impairment caused by ischemic stroke:

### Limb dysfunction:

Patients with ischemic stroke and intermittent claudication have both cerebral and peripheral atherosclerosis. Therefore, there is stenosis of the peripheral or anterior and posterior cerebral circulation artery lumen, resulting in a decrease in local cerebral blood flow, and then microcirculation disorders in the blood supply site. The metabolic rate of limb tissue in these patients is significantly increased. If the lumen stenosis reaches 50%, pressure changes on the flow gradient curve will occur, which will lead to insufficient blood perfusion in local muscle tissue, resulting in a series of pathological changes such as axonal degeneration, muscle atrophy and necrosis, which are ischemic brain diseases. Factors associated with intermittent claudication due to stroke.

More than 4.7 million patients with ischemic stroke have motor dysfunction as a sequela, and 30% to 66% of these patients have motor dysfunction in the upper limbs. Studies have shown that people with upper limb dysfunction for more than one year after stroke are often accompanied by more severe anxiety and lower quality of life and personal well-being. Compared with other limbs, the recovery of motor function of the upper limbs is a major difficulty that needs to be overcome in post-stroke rehabilitation treatment.

### Visual impairment:

Ischemic stroke may cause retinal ischemia, which is a leading cause of visual impairment and blindness worldwide. Since adequate blood supply to the retina is critical to maintaining its high metabolic demands, any obstruction to blood flow may result in reduced oxygen supply, leading to retinal ischemia.

In the pathogenesis of retinal ischemia, ischemic stroke leads to insufficient oxygen delivery to the retina and ultimately leads to visual impairment. Other potential causes of retinal ischemia include neuronal dysfunction due to environmental factors. The exact visual impairment caused by ischemic stroke varies between patients, so the progression remains unclear.

### Dysphagia:

As mentioned above, ischemic stroke is the most common type of cerebral infarction. It is caused by cerebral atherosclerosis, which causes thickening of blood vessel walls and narrowing of blood vessels, resulting in reduced blood flow or insufficient blood supply to the brain, causing local lesions in the nervous system. It is a common disease among middle-aged and elderly people, with the characteristics of high morbidity, disability, and mortality. Post-ischemic stroke dysphagia is also a disease recognized by the World Health Organization. It is significantly related to complications such as malnutrition, dehydration, and aspiration, resulting in poor patient prognosis and high mortality. Patients will still have sequelae after timely treatment. These situations will lead to prolonged hospital stay, increased mortality rate after discharge, and have a significant impact on the patient’s physical and mental health, and even endanger their lives.^27^

A large number of ischemic stroke patients will develop varying degrees of dysphagia, mainly manifested as difficulty in eating or drinking, repeated swallowing in small mouths, and dysphonia. According to literature reports, the incidence of dysphagia after stroke ranges from 19% to 81%.^28^ This range may be related to the assessment methods and diagnostic standards chosen by researchers, because there is a lack of unified clinical standards for the diagnosis and assessment of dysphagia, resulting in large differences in the results of the incidence of dysphagia. Among patients with ischemic stroke who have dysphagia but are conscious, the dysphagia seriously affects the patient’s eating function, which can easily lead to malnutrition, water and electrolyte disorders, and poor prognosis. About 33% of patients may die within six months of onset. Therefore, it is very necessary to take timely and effective measures to treat dysphagia patients with ischemic stroke.

The incidence of dysphagia after ischemic stroke is quite high.^29^ Mainly due to the damage to the coordination between the upper esophageal sphincter and the pharynx, incomplete relaxation of the upper esophageal sphincter during swallowing, progressive decrease in swallowing-related sensory functions, impaired pharyngeal sensation, and weakened gag reflex. Swallowing is a complex, subtle, and coherent movement controlled by multiple motor and sensory nerves. It is the finalization process of a sequence of actions.

The entire swallowing process can be divided into four stages. These four stages are the oral preparation stage, the oral stage, the pharyngeal stage, and the esophageal stage. During normal swallowing, the geniohyoid muscle, thyrohyoid muscle, digastric muscle, and other muscle groups contract, lift the hyoid bone, and shorten the distance between the larynx and hyoid bone. The epiglottis covers the trachea and the upper esophageal opening opens. Helps food bolus enter the esophagus smoothly. Any one of these abnormalities can cause dysphagia. The neural structures and mechanisms involved in dysphagia after ischemic stroke are very complex. In recent new studies, the concept of “sarcopenic dysphagia” has been proposed. It refers to the reduction of overall muscle mass in stroke patients, accompanied by changes in swallowing-related muscle groups, leading to weakened muscle strength, chewing weakness, muscle contraction disorders, and loss of muscle function, which hinders stroke rehabilitation and recovery of swallowing function.^30^

After the onset, the function of the brain tissue that controls the laryngeal muscles and respiratory muscles is damaged or nerve conduction is blocked, and the movement of the respiratory muscles and glottis closure cannot be performed. With effective coordination, dysphagia in the laryngeal stage is more harmful.^31^ At present, patients are often given swallowing training in clinical practice to improve their swallowing function. Avoid disuse atrophy or ataxia in patients’ swallowing-related muscle groups. Enhance the patient’s swallowing muscle strength and promote coordinated movement of the swallowing muscles to improve the patient’s swallowing ability and feeding ability. Reduce or avoid serious consequences such as malnutrition, dehydration, aspiration pneumonia, repeated lung infections, and suffocation. Thereby greatly improving the quality of life of patients.

### Rehabilitation treatment for dysphagia:

Swallowing is an important physiological function of the human body. The swallowing process is closely related to the cerebral cortex, brainstem nuclei, cranial nerves, and swallowing centers. The completion of the swallowing function is closely related to the oral cavity, esophagus, and throat. Swallowing disorder is a common complication of ischemic stroke.^31^,^32^ The patient’s swallowing function is impaired, which leads to malnutrition, affects the treatment effect, and causes respiratory infections. Observe the clinical symptoms of patients with neurogenic dysphagia and adopt corresponding treatment and nursing measures to improve the patient’s quality of life.

It is crucial to improve swallowing disorders in stroke patients through rehabilitation. Traditional rehabilitation methods such as swallowing training and electrical stimulation are widely used in clinical practice. Currently, there is no specific treatment for dysphagia after stroke. In clinical practice, gastric tube feeding is often used to prevent malnutrition or aspiration in patients with dysphagia. However, this method not only brings great pain to the patient, but also brings serious consequences to the patient’s family.

A huge burden. In recent years, swallowing rehabilitation training has been used clinically to improve swallowing function by training respiratory muscle strength combined with swallowing. With the advancement of science and technology, some potential neurological rehabilitation methods are also being developed. At present, the rehabilitation of patients with dysphagia after ischemic stroke mainly emphasizes early clinical intervention and intervention during rehabilitation treatment. The use of new technologies to restore swallowing movements in stroke patients can provide potential therapeutic strategies for future prevention.

### Clinical nursing care:

Aspiration due to dysphagia after stroke is a high-risk factor for pneumonia. Therefore, in clinical practice, for ischemic stroke patients with dysphagia, their swallowing function should be assessed promptly and accurately, and appropriate and targeted rehabilitation treatment should be implemented. Treatment measures also provide nutritional support. After the patient is admitted to the hospital, medical staff should take intervention measures against the risk factors of neurogenic dysphagia, control blood lipids, blood pressure, etc. After the patient’s vital signs stabilize, they should provide swallowing rehabilitation training to promote the recovery of the patient’s physiological functions, improve the prognosis and the patient’s quality of life. Adopt corresponding intervention measures for the factors of dysphagia after ischemic stroke.

Clinically observe the patient’s ability to cough spontaneously and clean the respiratory tract. If the patient has salivation, it indicates poor facial muscles. The hoarseness of the patient’s voice is used to observe the opening and closing function of the larynx. If the patient’s swallowing opening is significantly reduced and the mealtime is prolonged, it indicates that the patient has a swallowing disorder. Patients without the above clinical symptoms should be given a drinking test, repeated swallowing test, gag reflex test, etc. to evaluate the patient’s swallowing function.

At the same time, swallowing function can be evaluated with the help of equipment such as swallowing imaging video. Tube feeding is one of the important means of nutritional support for patients with dysphagia after stroke in clinical care, but this method has many shortcomings. For example, an indwelling gastric tube can cause damage to the patient’s gastrointestinal muscles, resulting in a weakened swallowing reflex. The gravity effect of the gastric tube can easily cause ulcers in the nasopharyngeal mucosa, which may promote the colonization of harmful bacteria in the oropharynx and easily cause aspiration pneumonia.^33^ These all affect the recovery of swallowing function. Therefore, nursing staff should regularly evaluate the swallowing function of patients using tube feeding, actively cooperate with swallowing rehabilitation training, and help patients extubate as soon as possible and resume independent eating. It is worth noting that endotracheal intubation or tracheotomy in the acute phase of stroke has a more significant adverse effect on the improvement of swallowing function. Recent studies have shown that most previous studies predicting the prognosis of swallowing function have excluded patients with tracheotomy, which may be to improve the operability of the study and the reliability of the results. And as mentioned above, invasive operations such as tube feeding may damage the muscles and nerves that control the swallowing process, leading to changes in tracheal biomechanics, which in turn lead to changes in the pharyngeal phase.^34^

### Recovery treatment:

Rehabilitation nursing intervention can significantly improve dysphagia after acute ischemic stroke. Systematic rehabilitation care can not only significantly improve the overall efficiency of functional recovery for patients with dysphagia, but also significantly enhance the coordination between patients’ swallowing muscles, increase the contractility of the tongue base muscles, and facilitate the development of patients’ swallowing reflexes. Clinical treatment of dysphagia is mostly a combination of several treatments, and single therapy often has poor efficacy.^35^

Nerve resection electrical stimulation is a major swallowing treatment method used clinically to treat dysphagia. It can stimulate the patient’s gradual transition from swallowing sensation to eventually participating in active swallowing and restore the patient’s active swallowing function. Repeated electrical stimulation and training of the patient’s swallowing muscles can promote or reconstruct the swallowing feedback pathway, reconstruct the swallowing motor center in the cerebral cortex, restore the patient’s nerve control and conduction functions, and thereby restore the patient’s swallowing function. Electrical stimulation can promote the recovery of related swallowing muscle strength. The current generated by electrical stimulation can induce passive muscle contraction, thereby avoiding the occurrence of disuse atrophy.^36^

Another commonly used nursing intervention plan is specialized swallowing ability training, feeding care, and psychological intervention.^38^ Swallowing ability training involves using a cotton swab treated with 2% glucose solution and frozen to stimulate the patient’s tongue base, causing the patient’s tongue base muscles to be stimulated and induced to contract. The patient’s throat function is then exercised by swallowing saliva and coughing out gas.

Feeding care is training to help patients master swallowing skills. During the feeding care process, the patient’s eating status needs to be observed in real time. Nursing staff should pay attention to the size, temperature and feeding speed of the food at all times. Finally, according to the patient’s emotional and psychological characteristics, a psychological intervention plan is formulated that suits the patient’s situation. Actively communicate with patients in daily care to keep patients optimistic and build confidence in treatment. Swallowing rehabilitation training can also improve the sensitivity of pharyngeal nerves, promote the formation of swallowing reflex, and delay the atrophy of pharyngeal muscles. In recent years, traditional Chinese medicine has also been studied as a method for treating dysphagia after stroke. Acupuncture, as part of a comprehensive treatment program of traditional Chinese medicine, is expected to help patients restore swallowing function more effectively in the future.^38^

For rehabilitation care of patients with dysphagia after stroke, most patients will choose subordinates or rehabilitation institutions to continue rehabilitation treatment or be discharged home. Home rehabilitation is mainly performed by informal caregivers.^39^ In 2024, Labeit B et al. published an article in The Lancet, elaborating on intervention strategies and treatments for dysphagia based on functional compensation and motor learning.^40^ Functional compensation refers to helping patients overcome dysphagia by adjusting the environment, tools or strategies to ensure that they can safely ingest food and liquids. Motor learning refers to improving the coordination and efficiency of swallowing movements through practice and training.^41^For patients with dysphagia after stroke, the combination of compensatory methods and motor learning methods can effectively promote the recovery of swallowing function, which can be achieved in a variety of ways. For example, choose food reasonably and control food dosage and viscosity. Use various swallowing techniques, movements and exercises during eating to strengthen the swallowing muscles and improve sputum secretion. And apply various nerve stimulations to swallowing-related muscles to increase prehyoid bone movement. Studies have shown that post-stroke dysphagia patients who choose hospitals for out-of-hospital care are more likely to fall into the high-risk group. It may be that the patient received treatment in the hospital in the early stage, and then the living environment changed and the family support he received decreased, which affected the patient’s recovery effect.^42^

Interviews with formal and informal nursing staff revealed that the best source of ideal care for patients is informal caregivers, that is, family caregivers.^43^Through follow-up studies, it was found that the family caregivers of post-stroke dysphagia patients recovering at home are mainly their spouses and children.^44^ Compared with medical staff, they can meet the emotional needs of patients. It provides patients with strong mental support, meticulous care, a harmonious family atmosphere and timely and effective psychological communication, so that patients can bravely face various problems that occur during the recovery process with a positive and optimistic attitude.

It helps patients face the disease, better cooperate with treatment and rehabilitation training, and promote recovery. Therefore, medical staff should start involving family caregivers in the patient’s care as soon as the patient is admitted to the hospital. Provide relevant disease knowledge education and care skills training to improve the care ability of family caregivers and promote the recovery of patients.^45^

## CONCLUSION

The world’s population is aging, and the incidence of stroke is expected to increase. Dysphagia is one of the common complications after ischemic stroke. A large proportion of stroke patients experience varying degrees of dysphagia. The patient’s main symptom is pharyngeal and sternal dysphagia during or within seconds after eating. Later, I felt a sticky or stagnant feeling and was unable to swallow normally. Dysphagia in patients with ischemic stroke can lead to symptoms such as malnutrition and severe dehydration. Malnutrition and dehydration can affect the nervous system, lead to complications such as aspiration pneumonia, and increase a patient’s risk of ischemic stroke. Disability and mortality rates. Therefore, it is urgent to find effective methods to treat dysphagia in patients with ischemic stroke. Swallowing is a complex reflex nerve activity. Individuals alone cannot accurately perceive the movement status of muscles. However, swallowing rehabilitation training can promote the recovery of swallowing muscles.

Orofacial and facial muscle training can enhance the function of type I muscle fibers in the swallowing system. The swallowing action is mainly performed by type II muscle fibers. Oral and facial muscle training has limited effectiveness. Muscle training such as cold stimulation can increase the sensory impulse awareness of the swallowing system, improve the sensitivity of the body’s central nervous system to swallowing movements, and enhance swallowing ability. The pursuit of lip breathing, and abdominal breathing is also helpful for rehabilitation and pulmonary function training in patients with ischemic stroke dysphagia.

We summarized the research progress on rehabilitation treatment programs for dysphagia in post-stroke patients. Conventional clinical rehabilitation measures for dysphagia mainly include sensory stimulation training, orofacial muscle function training, swallowing function training, muscle electrical stimulation training, etc. Swallowing rehabilitation training can help patients with swallowing disorders recover. With current technology, traditional conventional therapy remains the best treatment strategy. Dysphagia is a potentially treatable condition that, if successfully treated, will improve the patient’s subsequent quality of life. Furthermore, even though conventional therapies are empirically considered crucial to the recovery process, their effectiveness will be enhanced by further research into this technology, either used alone or in combination with various rehabilitation therapies to achieve positive results.
